# Effect of Electrical Contact Resistance on Measurement of Thermal Conductivity and Wiedemann-Franz Law for Individual Metallic Nanowires

**DOI:** 10.1038/s41598-018-23291-9

**Published:** 2018-03-20

**Authors:** Jianli Wang, Zhizheng Wu, Chengkun Mao, Yunfeng Zhao, Juekuan Yang, Yunfei Chen

**Affiliations:** 1 Jiangsu Key Laboratory for Design and Manufacture of Micro/Nano Biomedical Instruments, Southeast University, Nanjing, 210096 China; 2 Tianjin Key Laboratory of Advanced Functional Porous Materials, Institute for New Energy Materials & Low-Carbon Technologies, School of Materials Science and Engineering, Tianjin University of Technology, Tianjin, 300384 P.R. China

## Abstract

The electrical and thermal properties of metallic nanostructures have attracted considerable fundamental and technological interests. Recent studies confirmed a dramatic decrease in the electrical and thermal conductivities when the dimension is comparable or even smaller than the electron mean free path. However, the verification of the Wiedemann-Franz law in these nanostructures remains hotly debated. The Lorenz number obtained from the two-probe measurement is found to be much larger than that from the four-probe measurement. Here, we reported the electrical and thermal properties of the individual silver nanowires measured by the two-probe and four-probe configurations. The measured electrical contact resistance is found to be nearly temperature-independent, indicating a ballistic-dominant electronic transport at the contacts. When the effect of thermal contact resistance is diminished, the Lorenz number measured by the four-probe configuration is comparable to the Sommerfeld value, verifying that the Wiedemann-Franz law holds in the monocrystalline-like silver nanowire. Comparatively, the derived electrical conductivity becomes smaller and the thermal conductivity becomes larger in the two-probe measurement, confirming that the electrical contact resistance will introduce a large error. The present study experimentally demonstrates a reasonable explanation to the discouragingly broad span in the Lorenz number obtained from different metallic nanostructures.

## Introduction

The metallic nanowires have been successfully used in gas sensing, flexible touch screen and solar cells^1^. Their reduced size and high surface to volume ratio give rise to unique properties with respect to the bulk counterparts. Such phenomena are of considerable scientific and technological interests, thus have triggered a spate of theoretical and experimental works^2,3^.

Due to the difficulties in suspending a single nanowire and the risk of damages caused by the electrical impulses and the electrostatic, only a few experimental investigations have been reported to measure the thermal and electrical properties of the individual metallic nanowires^4–12^. The studies have led to a better understanding of charge and heat transport mechanisms in metallic nanowires. Consistent conclusions are drawn that the reduced size would give rise to a reduction in both thermal and electrical conductivities, when the dimension of metallic nanowire is comparable to or even smaller than the electron mean free path (MFP). However, the verification of the Wiedemann-Franz law on the metallic nanostructures has been hotly debating. The well-known electron-thermal analogy states that the ratio of the electronic thermal conductivity to the electrical conductivity at a given temperature equals to the universal Sommerfeld value, *LoT* = (π^2^/3)(*k*_B_/*e*)^2^*T* ≈ 2.44 × 10^−8^ W Ω K^−2^, where *k*_B_ and *e* are the Boltzmann constant and the magnitude of the electronic charge, respectively. The Wiedemann-Franz law applies to systems in which heat is predominantly transported by electrons, and holds to a good approximation when scattering is elastic. Ou *et al*.^6^ and Kojda *et al*.^11^ supported that this electron-thermal analogy is crudely valid for the nickel and silver nanowires at room temperature, while the significantly larger Lorenz numbers were also reported in silver, gold and platinum nanowires/nanofilms^4,9,10,12^. The discrepancy can be probably attributed to the differences in the material quality or/and the experimental techniques.

Noting that the self-heating method has been widely applied to measure the thermal and electrical conductivities of metallic nanowires/nanofilms, but the probe configurations are different in the measurements, both the two-probe (2-P) and four-probe (4-P) configurations were applied by different research groups. Interestingly, the Lorenz numbers obtained from the 2-P configuration^4,9,10,12^ are generally larger than those from the other^6,11^, and the values have a discouragingly wide span, ranging from 2.8 × 10^−8^ W Ω K^−2 ^^9^ to 1.3 × 10^−7^ W Ω K^−2 ^^10^. Even for the similar silver nanowire, the Lorenz number is obtained to be about 5.2 × 10^−8^ W Ω K^−2^ from the 2-P measurement^12^, but 2.2 × 10^−8^ W Ω K^−2^ from the 4-P measurement^11^, which is much closer to the Sommerfeld value. The silver nanowires used in the previous works^11,12^ may be different, so we cannot exclude the possibility that the large difference in the thermal and electrical properties of these nanowires is originated from the different material quality. Here, we compare the electrical and thermal properties of the individual silver nanowires measured by the 2-P and 4-P configurations, addressing the importance of the electrical contact resistance in the thermal property characterization.

## Results

**Figure 1:**
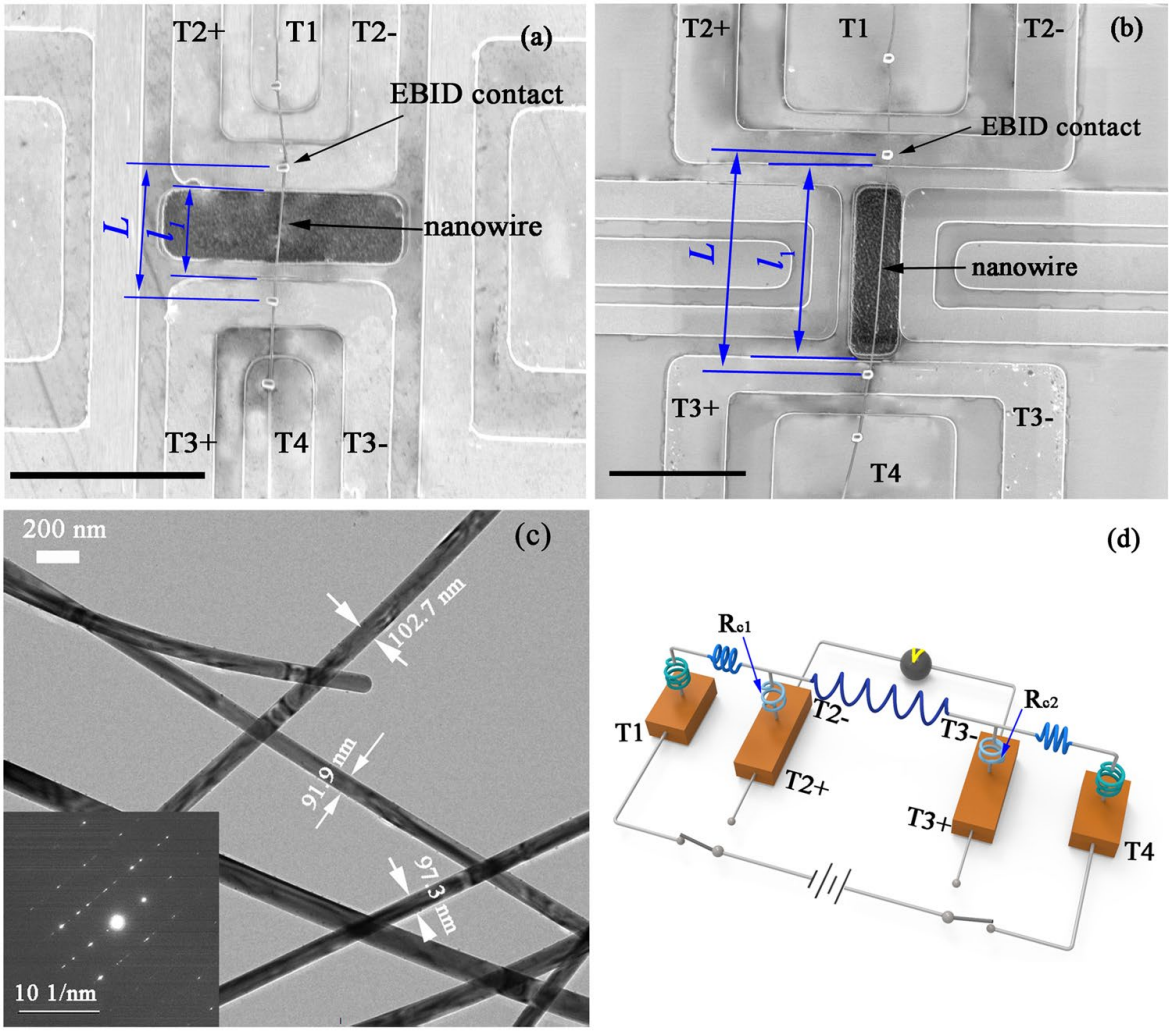
(**a,b**) SEM images of top-view of the free-standing silver nanowire samples. The lengths of the silver nanowires (*L*) are measured to be 6.49 μm (**a**) and 16.49 μm (**b**), while the suspended lengths (*l*_1_) are 4.88 μm (**a**) and 14.67 μm (**b**), respectively. The scale bar is 10 μm. (**c**) TEM image of the representative nanowires, the insert shows selected area electron diffraction patterns of the nanowires. (**d**) Schematic of electrical resistance circuit in the experiment. In the 2-P measurement, the direct current is imposed onto the terminals T2+ and T3+ , and the T2− and T3− terminals are connected to a digital multimeter. The electrical current is switched to terminals T1 and T2 to perform the 4-P measurement, so that the effect of the two electrical contact resistance *R*_c1_ and *R*_c2_ can be ignored.

**Figure 2:**
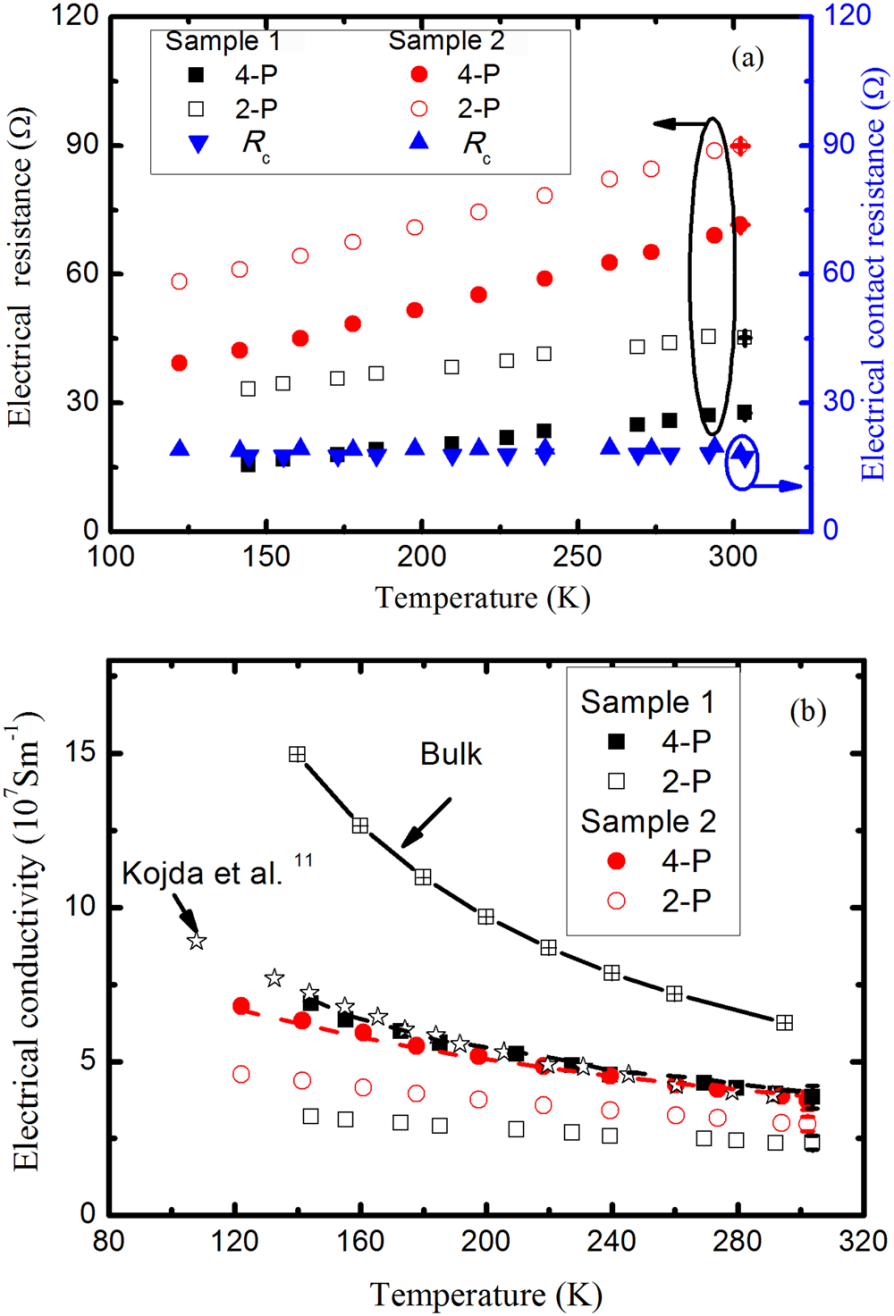
(**a**) Measured electrical resistance as a function of temperature, the derived electrical contact resistance is also shown with coordinate on the right, where *R*_c_ = *R*_c1_ + *R*_c2_. (**b**) Calculated electrical conductivity as a function of temperature. Bulk values^22^ are shown together with the reference value of silver nanowire with 150 nm diameter^11^.

**Figure 3:**
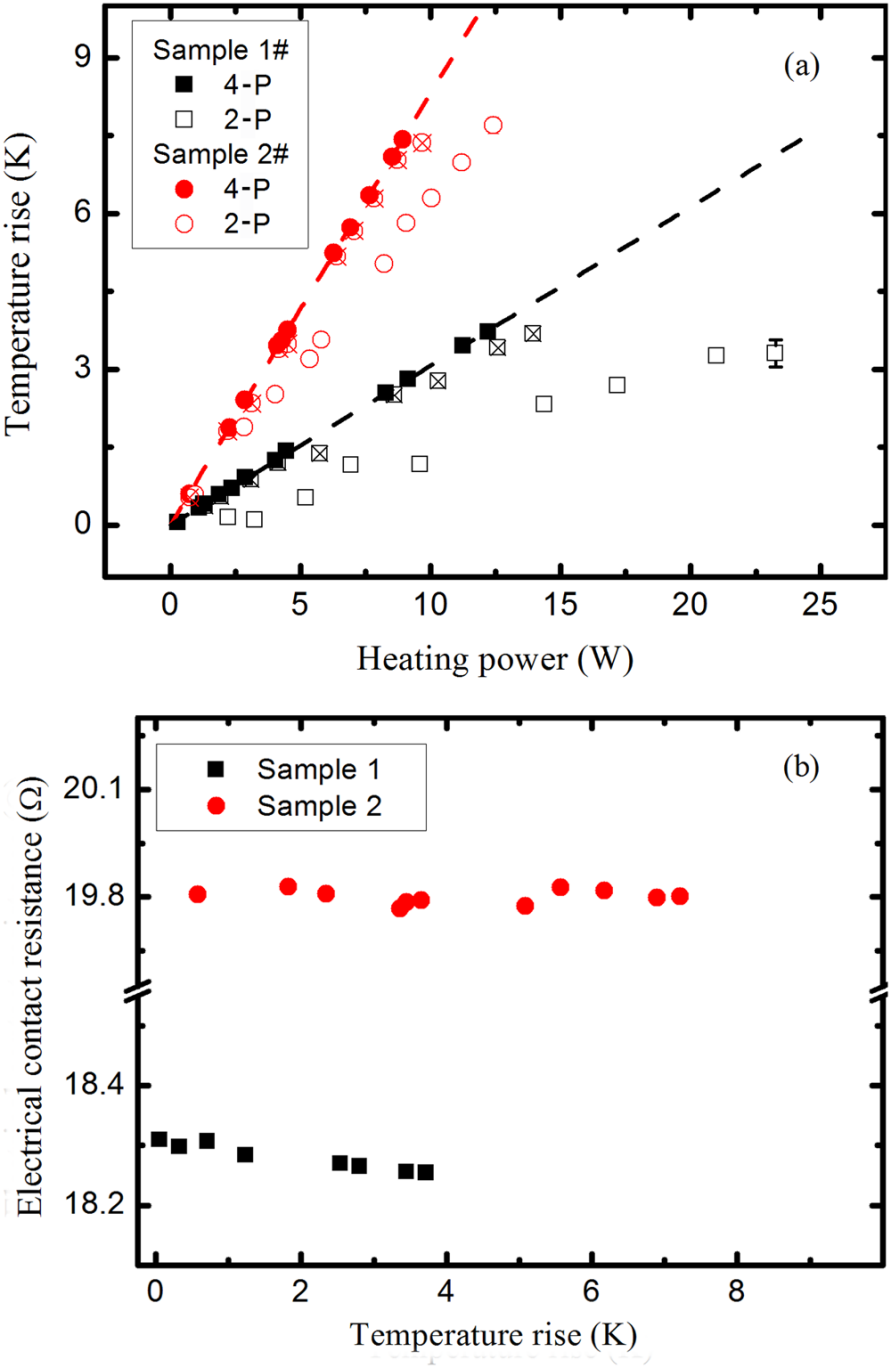
(**a**) Temperature rise as a function of heating power. The solid symbols are measured from the 4-P configuration, the blank symbols are for the 2-P configuration, and the cross symbols are corrected for the electrical contact resistance. (**b**) Calculated electrical contact resistance as a function of temperature rise.

Silver nanowires are synthesized by reducing silver nitriate (AgNO_3_) with ethylene glycol (EG) in the presence of poly(vinylpyrrolidone) (PVP)^13^. More details in the Methods section for the synthesis of nanowire. Two silver nanowires with different lengths are measured to clarify the end effect, and the geometries are presented in Table 1. Figure 1a and b shows the scanning microscope (SEM) image of the suspended silver nanowire. The nanowires have no obvious kinks in the suspended section, therefore, there are negligible few grain boundaries perpendicular to the growth direction. Figure 2a shows the temperature dependent electrical resistance of the two samples, the extracted electrical conductivity is shown in Fig. 2b. The electrical conductivities of the two samples from the 4-P configuration have similar values, confirming the reliability of measurement. In the temperature range 100 to 300 K. The electrical resistances from both the 2-P and 4-P configurations increase linearly as temperature increases, and the two curves are almost in parallel, so the derived electrical contact resistance is nearly temperature-independent. For the contacts enhanced by the electron-beam-induced deposition (EBID), the electrical contact resistances at two ends *R*_c_, i.e. *R*_c_ = *R*_c1_ + *R*_c2_, are found to be about 18 ± 0.5 Ω and 19 ± 0.5 Ω for sample 1 and sample 2, respectively. This phenomenon can be further verified in Fig. 3b, where the electrical contact resistance is nearly independent of the imposing currents.

**Table 1:** Summary of room temperarure property of silver nanowires. The thermal conductivity is calculated using Eq. (3).

Samples	Methods	*L* (μm)	*l*_1_ (μm)	*D* (nm)	*β* (10^−3^ K^−1^)	*σ* (10^7^ Sm^−1^)	*λ* (W m^−1^K^−1^)	*Lo* (10^−8^ WΩ K^−2^)
Sample 1	2-P	6.49	4.88	93.2	1.78	2.10	513	8.32
	4-P				2.96	3.51	285	2.85
Sample 2	2-P	15.49	14.67	97.0	2.11	2.36	273	3.87
	4-P				2.65	3.04	206	2.33
Chen *et al*.^12^	2-P	27.23	27.23	227	2.19	1.26	191.5	5.20
Kojda *et al*.^11^	4-P	11.00	~9	107	2.86	3.50	220	2.20

The Maxwellian contact resistance is given by *R*_c_ = 1/2*aσ*_*e*_, where *a* is the contact radius, *σ*_*e*_ is the effective electrical conductivity, which is calculated by *σ*_*e*_^−1^ = *σ*^−1^ + *σ*_Au_^−1^, *σ* and *σ*_Au_ are the electrical conductivity of the silver nanowire and the gold electrode, respectively. *σ*_Au_ and *σ*_*e*_ are shown in Figs S1 and S2 in Supporting Information. Since *a* can be treated as a constant value, the temperature dependent of the Maxwellian contact resistance should also follow the same trend as *σ*_*e*_, which is completely different from our findings. When the electron MFP of silver nanowire Λ is comparable with or even larger than *a*, the ballistic transport of electrons through the contacts cannot be ignored. The ballistic transport can be characterized by the Knudsen number, defined by *K* = Λ/*a*. For *K* ≫ 1, the Sharvin contact resistance can be crudely estimated by^14^

1 $${R}_{c}=\frac{4\Lambda }{3\pi {a}^{2}\sigma }=\frac{C}{{a}^{2}}$$

Λ/*σ* can be taken as a constant value over the temperature range of interest here, so the Sharvin contact resistance is also essentially temperature independent. For silver nanowire, *C* = 3.59 × 10^−16^ Ω m^2^, which is nearly the same to the value of gold electrode (3.54 × 10^−16^ Ω m^2^), thus we solely consider the silver nanowire side afterwards. Taken *R*_c1_ = *R*_c2_ = *R*_c_/2, *a* are estimated to be about 6.3 nm and 6.1 nm for sample 1 and sample 2, respectively. The MFP of silver nanowire decrease as temperature increases, see in Fig. S3 in Supplementary Information. The room temperature Λ ~ 34 nm, and the corresponding *K* is larger than 5 in both cases, confirming that the ballistic transport dominants in the nanowire/electrode contacts.

In the direct heating method, the metallic nanowire also serves as a resistance thermometer. The temperature coefficient of resistance (TCR) is defined by *β* = 1/*R*_*r*_(d*R*/d*T*), where *R*_*r*_ is the electrical resistance at reference temperature 273 K. The TCRs are obtained from Fig. 2a, and the values are listed in Table 1. The measured TCR is reduced with respect to the bulk counterparts, similar to the observations reported elsewhere^6,12,13,15^. Compared with that obtained from the 4-P measurement, the TCR from the 2-P measurement is expected to be smaller due to larger *R*_*r*_. After imposing a set of small currents, the temperature rise of silver nanowire, normally less than 10 K, can be calibrated from electrical resistance

2 $$\overline{{\rm{\Delta }}T}=\frac{{R}_{T}-{R}_{0}}{\beta {R}_{r}}$$

where *R*_0_ corresponds to the thermal resistance with zero heating power. The heat transfer through the test nanowire can be simplified to one-dimensional steady-state heat conduction. When the thermal resistance between the supported nanowire and the electrode is infinitely large, the thermal conductivity can be determined by

3 $$\lambda =\frac{{I}^{2}{R}_{T}L}{12S\overline{{\rm{\Delta }}T}}$$

where *L* and *S* are the length and cross-sectional area of nanowire, respectively. Derivation details can be found in Methods section. The average temperature rise of nanowire is found to increase linearly with the heating power, as shown in Fig. 3a. A large difference exists in the slope obtained from the 2-P and 4-P measurements. We can proof that this difference is originated from the temperature-independent electrical contact resistance, as observed in Fig. 3b. According to the physical model of the one-dimensional steady-state heat conduction, *R*_T_ in Eq. (3) is the electrical resistance of the nanowire with length *L*, and the contact resistance at the ends, *R*_c1_ and *R*_c2_ as denoted in Fig. 1d, should be excluded. Based on this fact, *R*_T_ from the 2-P configuration is corrected by subtracting *R*_c_, then the results are found to be nearly consistent with that from the 4-P measurement, as shown in Fig. 2a, indicating that the Joule heating and thermoelectric cooling^16^ at contacts can be ignored.

**Figure 4:**
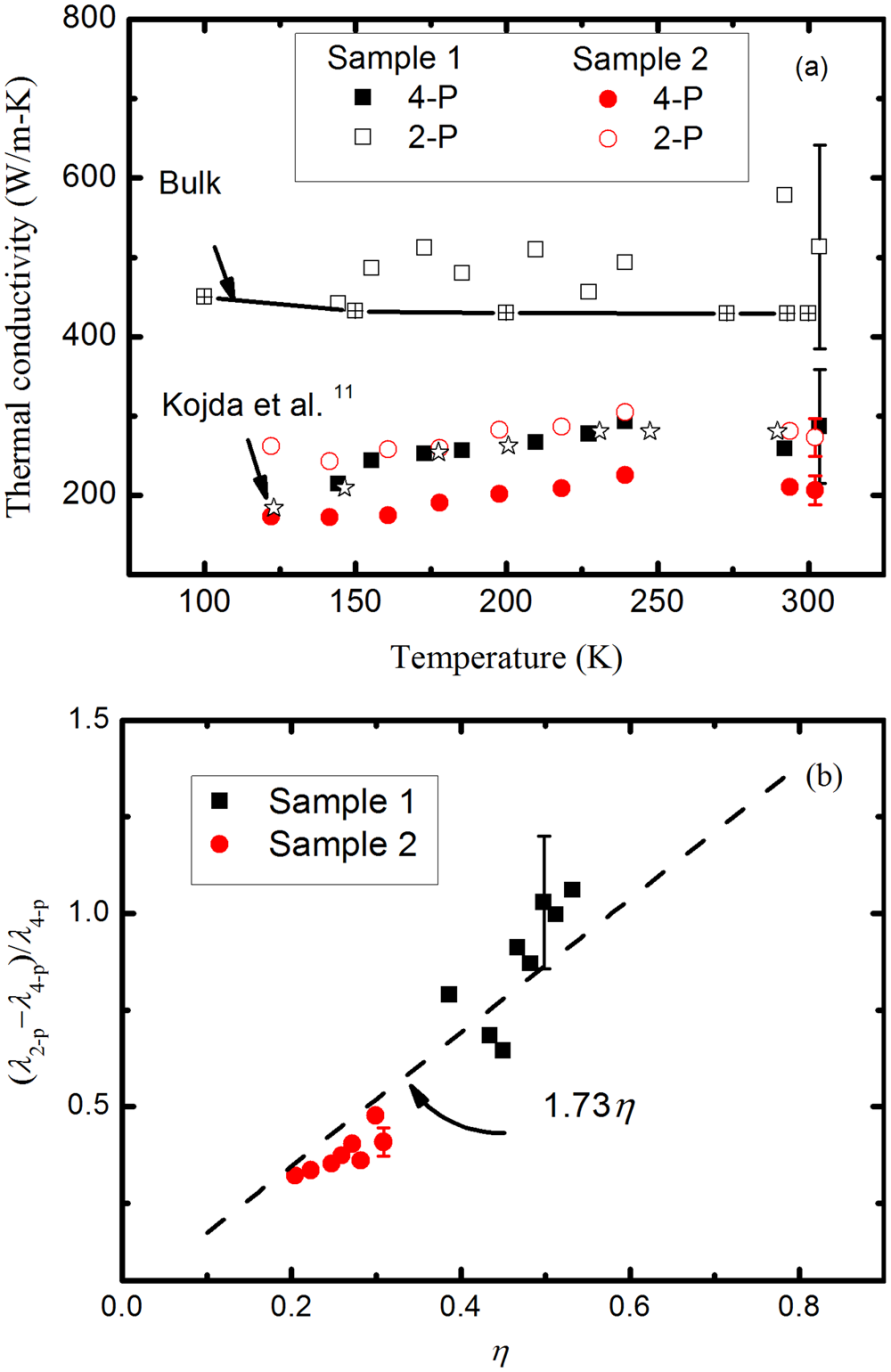
(**a**) Extracted thermal conductivity as a function of temperature. (**b**) Difference of thermal conductivities obtained from the 2-P and 4-P configurations with respect to the ratio of the electrical contact resistance to the total electrical resistance, the dashed line is the prediction from the accumulative error function.

Figure 4a shows the extracted thermal conductivities as a function of temperature. The thermal conductivities obtained from the 2-P configuration ranges from 440 to 580 W m^−1^ K^−1^ for sample 1 and ranges from 240 to 300 W m^−1^ K^−1^ for sample 2, which are significantly larger than the values from the 4-P measurement. It is worth noting that sample 1 is much shorter than the sample 2, therefore, the electrical contact resistance would play a more significant role in the 2-P measurement, resulting in a much larger thermal conductivity. We further evaluate the main sources of error given by the 2-P measurement. Figure 4b plots the relative difference of thermal conductivity, defined by (*λ*_2-p_ − *λ*_4-p_)/*λ*_4-p_, as a function of the ratio of contact resistance to the measured electrical resistance, *η* = *R*_c_/*R*_T_. Combined with Eqs (2) and (3), the measurement error of thermal conductivity from the 2-P configuration can be estimated by the accumulative error function

4 $$\frac{{\lambda }_{2 \mbox{-} p}-{\lambda }_{4 \mbox{-} p}}{{\lambda }_{4 \mbox{-} p}}=\sqrt{{(\frac{{\rm{\Delta }}{R}_{T}}{{R}_{T}})}^{2}+{(\frac{{\rm{\Delta }}\beta }{\beta })}^{2}+{(\frac{{\rm{\Delta }}{R}_{r}}{{R}_{r}})}^{2}}$$

where Δ*R*_T_, Δ*β* and Δ*R*_*r*_ are the differences of the electrical resistance, TCR and electrical resistance at reference temperature of the nanowire obtained from the 2-P and 4-P configurations. Based on the fact that the electrical resistance obtained from the two configurations are almost in parallel (Fig. 2a), and the difference in d*R*/d*T* can be neglected, therefore, we can have Δ*β*/*β* ≈ Δ*R*_*r*_/*R*_*r*_. Considering Δ*R*_T_ = *R*_c_ and Δ*R*_*r*_/*R*_*r*_ ≈ Δ*R*_T_/*R*_T_, the accumulative error is estimated to be (*λ*_2-p_ − *λ*_4-p_)/*λ*_4-p_ ≈ 1.73*R*_c_/*R*_T_. Figure 4b shows that the relative difference in thermal conductivity obtained from the two configurations can be well predicted by the accumulative error function. This consistency confirms that the electrical contact resistance plays a dominant role in the 2-P configuration in determining the thermal conductivity of metallic nanowire.

Compared with the small difference in the electrical conductivities of the two samples with different lengths (Fig. 2b), Fig. 4a shows that the thermal conductivity of the short sample from the 4-P measurement is about 20% larger than the long one, which is completely different with the length-dependent thermal conductivity in carbon nanotubes^17^ and graphene^18^. The thermal conductivity increases with length in these nanomaterials due to the quasi-ballistic thermal transport, when the MFP is comparable to the length. However, the MFP of silver nanowire is less than 100 nm (Fig. S3 in Supplementary Information), so the heat conduction along the nanowire should be dominated by the diffusive transport. One possible explanation is that the thermal conductivities from the short sample can be over-estimated. If the thermal resistance between the supported nanowire and the electrode is so small that the parabolic temperature profile is established solely in the suspended segment of nanowire, Eq. (3) should be changed to

5 $$\lambda =\frac{{I}^{2}{R}_{T}{{l}_{1}}^{3}}{12S{L}^{2}\overline{{\rm{\Delta }}T}}$$

where *l*_1_ is the length of the suspended segment of nanowire, as shown in Fig. 1a. In this case, the thermal conductivity of sample 1 from the 4-P measurement is found to be over 30% smaller compared with that of sample 2, and both values are smaller than the published data of silver nanowire with 150 nm diameter^11^. The two samples have similar diameter of about 100 nm, the corresponding thermal conductivity is also expected to the same. By measuring the two nanowires with different lengths, the thermal resistance per unit length (at room temperature) between the supported nanowire and the electrode can be estimated to be about 0.2 m K W^−1^, which is much smaller than the reported value between the carbon nanotube and substrate^19–21^. The impact of thermal resistance between the supported nanowire and the electrodes on the thermal conductivity measurement is analyzed in Supplementary Information.

From the electrical and thermal properties discussed above, in the 2-P measurement, we notice that the derived electrical conductivity would be smaller while the thermal conductivity would be larger, thus gives rise to a significant larger Lorenz number, which is defined by

6 $$Lo=\frac{\lambda }{\sigma T}$$

**Figure 5:**
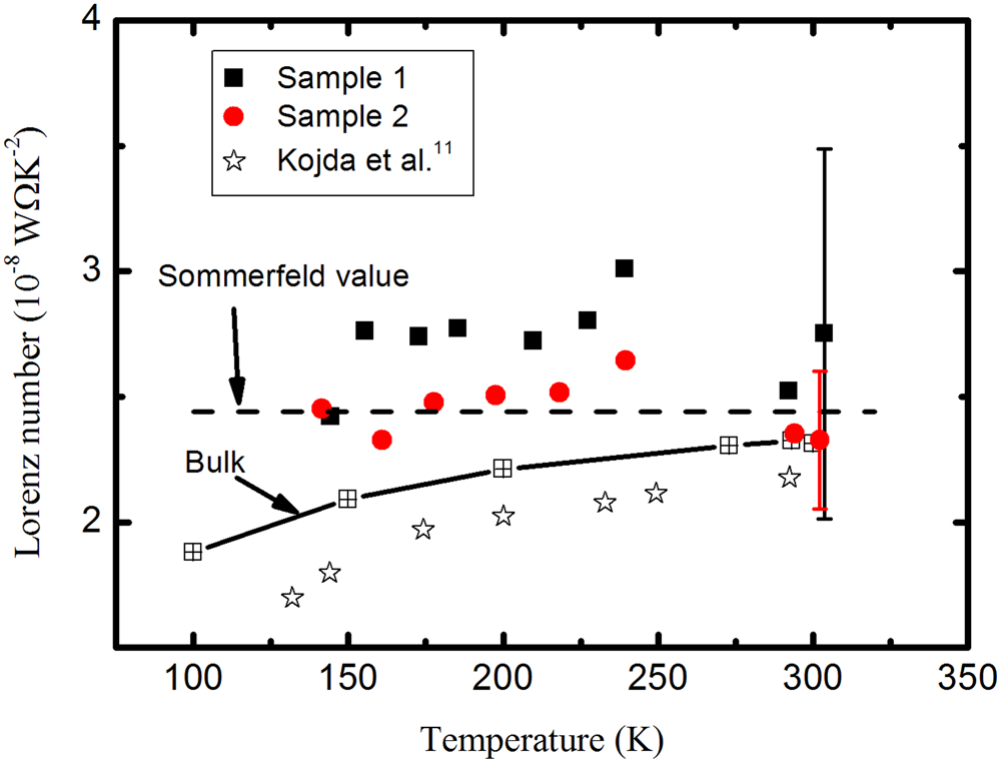
Measured Lorenz number as a function of temperature.

With the thermal conductivity calculated from Eq. (3), the Lorenz numbers obtained from the 2-P configurations are at around 9.5 ± 2.5 × 10^−8^ W Ω K^−2^ (sample 1) and 5.0 ± 0.6 × 10^−8^ W Ω K^−2^ (sample 2), respectively. Comparatively, according to the results obtained from the 4-P configuration, the Wiedemann-Franz law is generally valid particular for the long sample when the end effect in determining the thermal conductivity can be diminished, as shown in Fig. 5. As a result, we can safely draw a conclusion that the Wiedemann-Franz law holds for the monocrystalline-like silver nanowire. However, for the nanocrystalline metallic nanowire/nanofilm, when the grain size is comparable or even smaller than the MFP, the grain boundary might exert different influences on the charge and electronic thermal transport^4^, the validity of the Wiedemann-Franz law in this system needs further verification.

## Conclusions

The electrical and thermal properties of two silver nanowires with different lengths are measured using the 2-P and 4-P configurations. The obtained electrical resistances at the EBID-enhanced contacts have a similar value, and are nearly temperature-independent, indicating that the electrons transport through the contacts in a ballistic behavior. In the 2-P measurement, with the increase of the ratio of the electrical contact resistance to the measured electrical resistance, the derived electrical conductivity becomes smaller while the thermal conductivity is larger, resulting in a significant larger Lorenz number. The results from the 4-P measurement confirm that the Wiedemann-Franz law holds for the monocrystalline-like silver nanowire. Subsequently, the present study provides a reasonable explanation to the discrepancy in the electrical and thermal characterization of the metallic nanowire.

## Methods

### Synthesis of silver nanowire

During the production process, the silver nanoparticles firstly grow into multitwin particles. As the addition of Ag^+^ ions, the multiwin particles further grow into nanowires. In a typical experiment, 10 mL of EG is heated with magnetic stirring in an oil bath at 160 °C for 10 mins, then 6 mL of 0.5 mM AgNO_3_/EG solution and 6 mL of 0.75 mM PVP/EG solution are simultaneously added to the solution under magnetic stirring by a two-channel syringe-pump at an injection speed of 0.375 mL/min. Next, the solution is kept in the oil bath for another 4 hrs. The final product is obtained by centrifugation to remove the excess PVP and is dispersed in ethanol for further characterization. The electron diffraction confirms the presence of fcc structure, and the silver nanowire grows along the [110] direction, as shown in inset of Fig. 1c. A representative TEM image of the synthesized nanowires is given in Fig. 1c.

### Electrical characterization

Four gold electrodes with thickness of about 100 nm are deposited onto a thermal oxide silicon substrate with a 5 nm thick Cr film as an adhesion layer. The inner two electrodes are connected with four terminals, denoting as T2+ , T2−,T3+ and T3− (Fig. 1a and b), and the gaps between the two electrodes are of about 5 μm, and 15 μm, respectively. The reactive ion etching (DRIE) is used to trench the gap to a depth of about 1 μm. A probe station is used to manipulate a homogenous nanowire and bridge it across the two electrodes. The platinum/C is deposited to make stable electrical contacts between the electrodes and nanowire (FEI Helios 600i).

In the 2-P configuration, a small direct electrical current (100 nA) is imposed on the T2+ and T3+ terminals, while the voltage drop between the T2− and T3− terminals is recorded by a digital multimeter (Agilent 3458 A) with input impedance of over 10 GΩ, so the T2− and T3− terminals draw little current. For the same silver nanowire, the electrical current is switched to the T1 and T4 terminals to perform the 4-P experiment. Obviously, in the 4-P configuration, the electrical contact resistances between nanowire and electrodes, denoting as *R*_c1_ and *R*_c2_ in Fig. 1d, would be excluded in the detected electrical resistance of the nanowire.

### Thermal conductivity characterization

The thermal conductivity of the nanowire is measured by the direct-heating method^4,12^. The experiment is carried out under a high vacuum chamber with pressure less than 10^−4^ Pa at a set temperature ranging from 100 to 300 K, so that the residual gas molecular conduction and convection can be suppressed. Figure 1a and b shows that the test nanowire between the two inner EBID-enhanced contacts (length *L*) can be divided into two segments, one is supported by the electrode, and the other is suspended (length *l*_1_). If the thermal resistance between the supported nanowire and the electrode is infinitely large, and the two EBID-enhanced contacts are maintained at a set temperature *T*_0_, as a direct current *I* flows through the nanowire, the one-dimensional steady-state heat transfer governing equation is

7 $$\frac{{d}^{2}{\rm{\Delta }}T(x)}{d{x}^{2}}+\frac{{I}^{2}{R}_{T}}{\lambda LS}=0$$

Then the temperature rise along the nanowire can be obtained, which is expressed by

8 $${\rm{\Delta }}T(x)=-\frac{{I}^{2}{R}_{T}}{2\lambda SL}{x}^{2}+\frac{{I}^{2}{R}_{T}}{2\lambda S}x$$

Finally, the average temperature rise of the nanowire is

9 $$\overline{{\rm{\Delta }}T}=\frac{1}{L}{\int }_{0}^{L}{\rm{\Delta }}T(x)dx=\frac{{I}^{2}{R}_{T}L}{12\lambda S}$$

From Eq. (9), the average temperature rise should increase linearly with *I*^2^*R*_T_. In the 2-P measurement, the measured electrical resistance from the T2− and T3− terminals in Fig. 1d, *R*_*M*_, includes the effect from the electrical contact resistances at the two ends, therefore, the average temperature rise should be

10 $$\overline{{\rm{\Delta }}T}=\frac{{I}^{2}({R}_{M}-{R}_{c})L}{12\lambda S}$$

Our results show that *R*_c_ is temperature independent, so the average temperature rise in the 2-P measurement also follows linearly with *I*^2^*R*_*M*_, as shown in Fig. 3a.

## Electronic supplementary material

Supplementary Information
