# Supplementary material for: Effect of Electrical Contact Resistance on Measurement of Thermal Conductivity and Wiedemann-Franz Law for Individual Metallic Nanowires
Source: Sci Rep. 2018 Mar 20;8:4862. doi: 10.1038/s41598-018-23291-9 (PMC5861060; doi:10.1038/s41598-018-23291-9)
Supplement: Supplementary file 1 — Supplementary Information [file 41598_2018_23291_MOESM1_ESM.docx]

**Supplementary Information**

Effect of Electrical Contact Resistance on Measurement of Thermal Conductivity and Wiedemann-Franz Law for Individual Metallic Nanowires

Jianli Wang^1,*^, Zhizheng Wu1, Chengun Mao1, Yunfeng Zhao2, Juekuan Yang1 & Yunfei Chen1

1 Jiangsu Key Laboratory for Design and Manufacture of Micro/Nano Biomedical Instruments, Southeast University, Nanjing 210096, China

2 Tianjin Key Laboratory of Advanced Functional Porous Materials, Institute for New Energy Materials & Low-Carbon Technologies, School of Materials Science and Engineering, Tianjin University of Technology, Tianjin 300384, P.R. China

^a)^ Author to whom correspondence should be addressed;

Tel: +86-(0)25-52090501; Fax: +86-(0)25-52090504

E-mail address: [wangjianli@seu.edu.cn](mailto:wangjianli@seu.edu.cn) (J. W.)

**Effective electrical conductivity at the gold/silver contact**

The gold film with thickness of 100 nm is deposited onto a substrate in the same deposition condition as that applied in fabricating the silicon device used in this work. The gold film is further patterned into the four-probe configuration using the photolithographic technique with 5 mm length and 20 μm width, as shown in inset of Fig. S1. The electrical conductivity of gold electrode is shown in Fig. S1, in comparison with the reference data. To calculate the Maxwellian contact resistance, the effective electrical conductivity *σ_e_* is estimated by

| $\frac{1}{\sigma_{e}}=\frac{1}{\sigma}+\frac{1}{\sigma_{Au}}$ | (S1) |
| --- | --- |

Combined with the electrical conductivity of silver nanowire shown in Fig. 2b in manuscript, the temperature dependent of *σ_e_* for sample 1 and sample 2 is plotted in Fig. S2. The Maxwellian contact resistance is expressed by

| $R_{c}=\frac{1}{2a\sigma_{e}}$ | (S2) |
| --- | --- |

The contact radius *a* is taken to be temperature-independent, so the Maxwellian contact resistance should follow the same trend as the reciprocal of *σ_e_*, which is completely different from our findings.

| 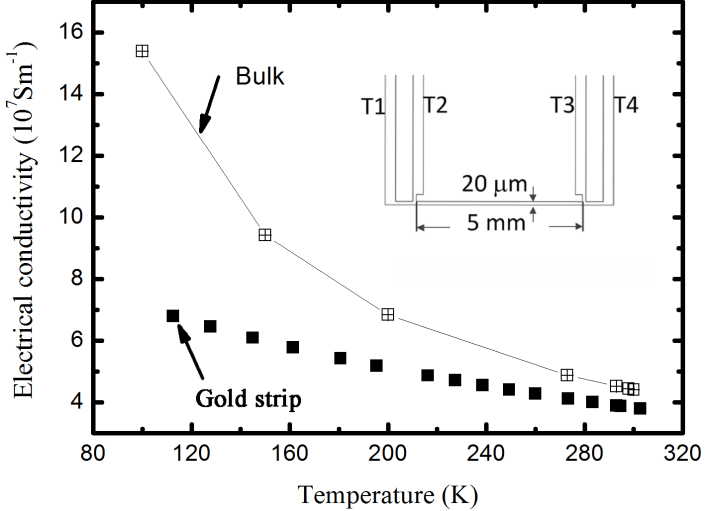 |
| --- |
| Figure S1. Electrical conductivity of gold electrode as a function of temperature, the inset shows the shape of the gold strip. |

| 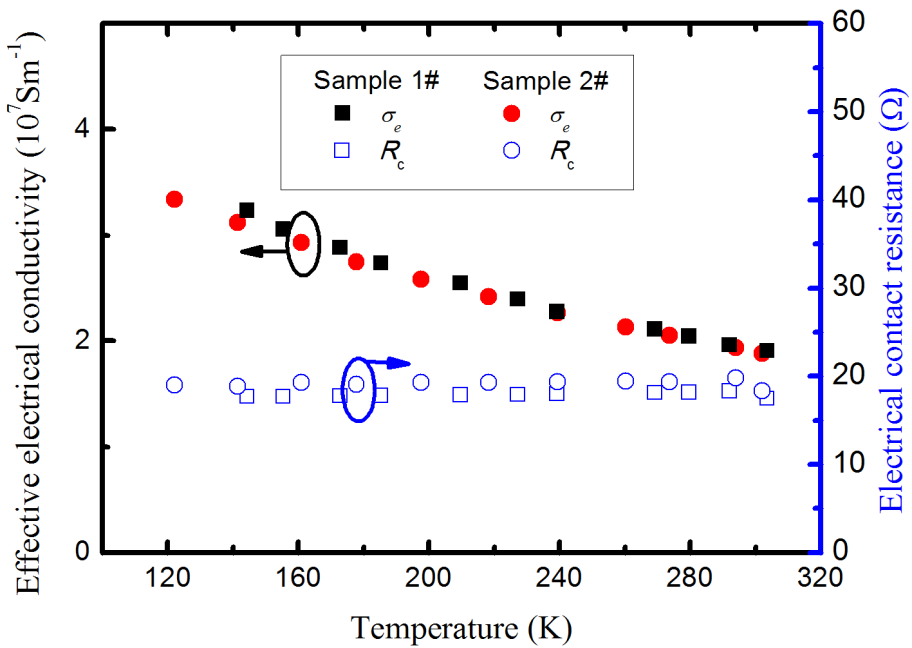 |
| --- |
| Figure S2. Effective electrical conductivity as a function of temperature, and the derived electrical contact resistance is shown with coordinate on the right. The effective electrical conductivity decreases as temperature increases, while the electrical contact resistance is nearly temperature-independent. |

**Mean free path of gold electrode**

The mean free path of bulk gold Λ*_b_* is 37.7 nm, and the electrical conductivity *σ*_Au,b_ is 4.516×10^7^ S m^-1^ ^S1^. Taking the bulk value of electrical conductivity, the temperature dependent of the MFP in gold electrode can be estimated by Λ_f_/*σ*_Au,f_=Λ*_b_*/*σ*_Au,b_, where Λ_f_ and *σ*_Au,f_ are the MFP and electrical conductivity of the gold film, respectively. Λ_f_ is plotted as a function of temperature in Fig. S3. Similar to the results from the silver nanowire, the MFP of the gold electrode is also expected to be much larger than the contact radius *a*.

| 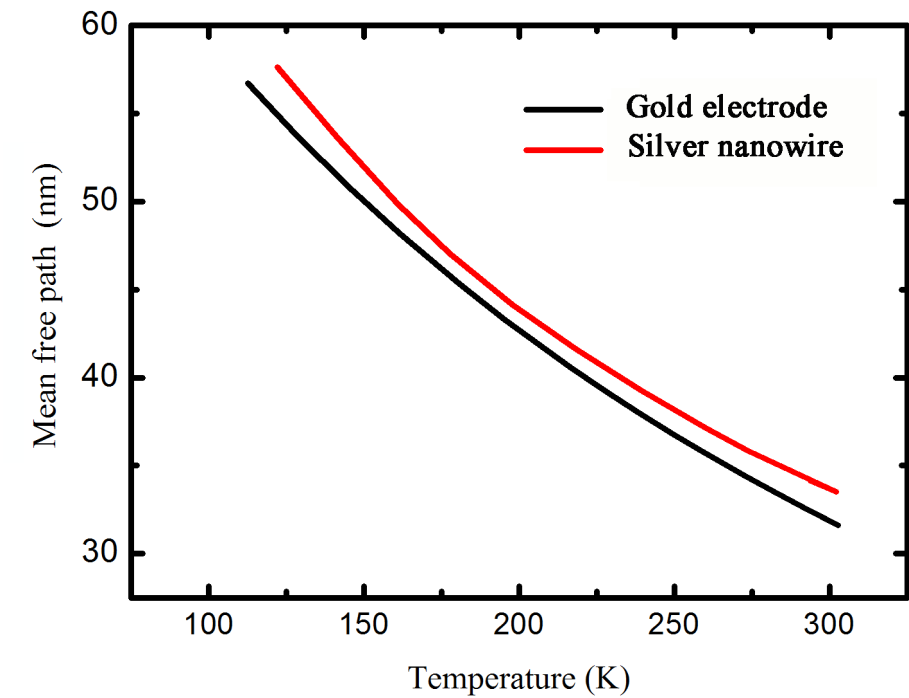 |
| --- |
| Figure S3. Electron mean free path of the gold electrode and the silver nanowire as a function of temperature. |

**Thermal resistance between supported nanowire and electrodes**


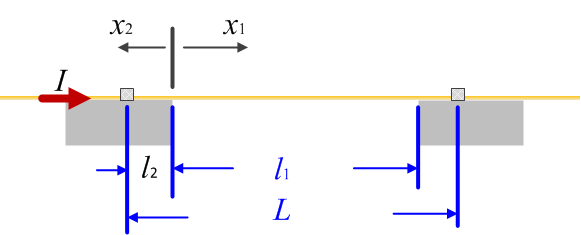


Figure S4. Schematic of the suspended Ag nanowire with EBID-enhanced contacts.

The SEM images in Fig. 1a and 1b show that the test nanowire between two inner EBID-enhanced contacts can be divided into two segments, one is supported by the electrode, and the other is suspended. With the coordination illustrated in Fig. S4, the heat governing equation along the test nanowire can be expressed by:

For the suspended segment

$\frac{d^{2}\Delta T}{dx_{1}^{2}}+\frac{I^{2}R_{T}}{\lambda LS}=0$ (S3)

For the supported nanowire ^S2^

$\frac{d^{2}\Delta T}{dx_{2}^{2}}-\frac{\Delta T}{\lambda SR_{S}}+\frac{I^{2}R_{T}}{\lambda LS}=0$ (S4)

where *L*=*l*_1_+2*l*_2_, *l*_1_ and *l*_2_ are the suspended length and the contact length of nanowire at one electrode, respectively. *R*_T_ is the electrical resistance of the test nanowire with length *L*, *R*_s_ is the thermal resistance per unit length between the supported nanowire and the electrode. Finally, the temperature distribution along the test nanowire can be obtained by

| $\overline{\Delta T}=\frac{I^{2}R_{T}}{\lambda LS}\left\{\frac{1}{b^{2}}+\frac{1}{12}\frac{l_{1}^{3}}{L}+\frac{1}{b^{2}}\frac{l_{1}}{L}+\frac{1}{\cosh(bl_{2})}\frac{l_{1}}{L}\left[-\frac{2}{b^{2}}+\sinh(\alpha l_{2})\left(\frac{l_{1}}{2b}-\frac{2}{b^{3}l_{1}}\right)\right]\right\}$ | (S5) |
| --- | --- |

with *b*^2^=(*λSR_s_*)^-1^. Obviously, Eq. (S5) is reduced to Eq. (5) in manuscript when *R*_s_ is infinitely small.

The suspended and contact lengths of nanowire are found to be *l*_1_=4.89 μm, *l*_2_=0.80 μm for sample 1 and *l*_1_=14.67 μm, *l*_2_=0.41 μm for sample 2, respectively. Supposed that *R*_s_ is the same in the two measurements, by measuring the two nanowires with different lengths using the 4-P configuration, *R*_s_ can be crudely estimated based on Eq. (S5). Fig. S5 shows the extracted thermal conductivity when *R*_s_ is taken to be 0.2 m K W^-1^, and the corresponding Lorenz numbers are shown in Fig. S6. Compared with the results presented in Fig. 4a in manuscript and Fig. S5, we confirm that the end effect has a significant effect in determining the thermal conductivity, especially for the short nanowire.


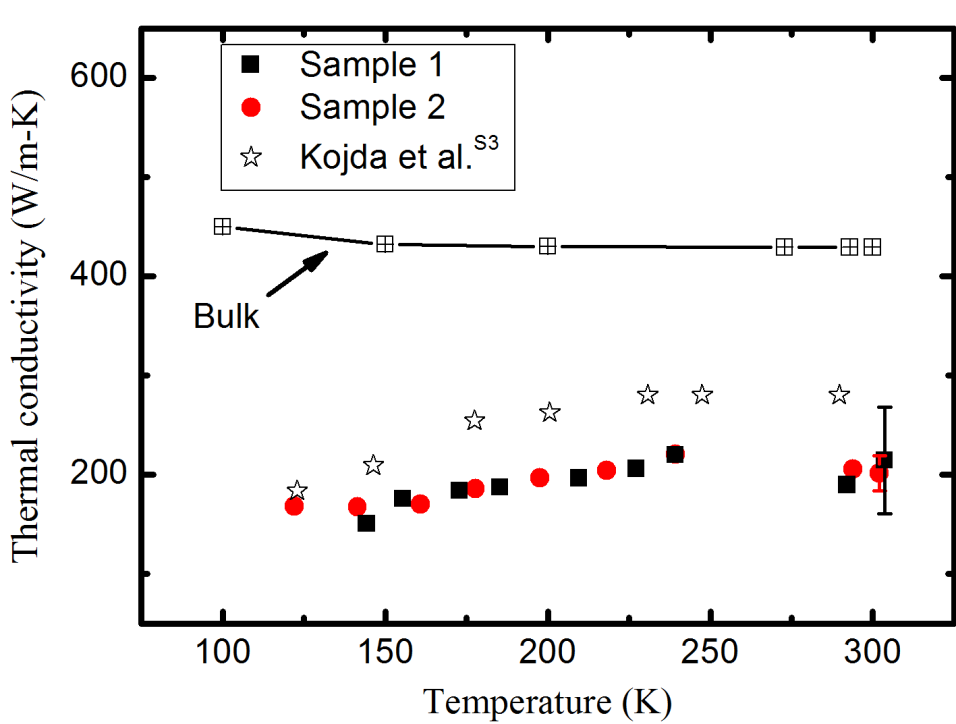


Figure S5. Extracted thermal conductivity as a function of temperature when *R*_s_=0.2 m K W^-1^


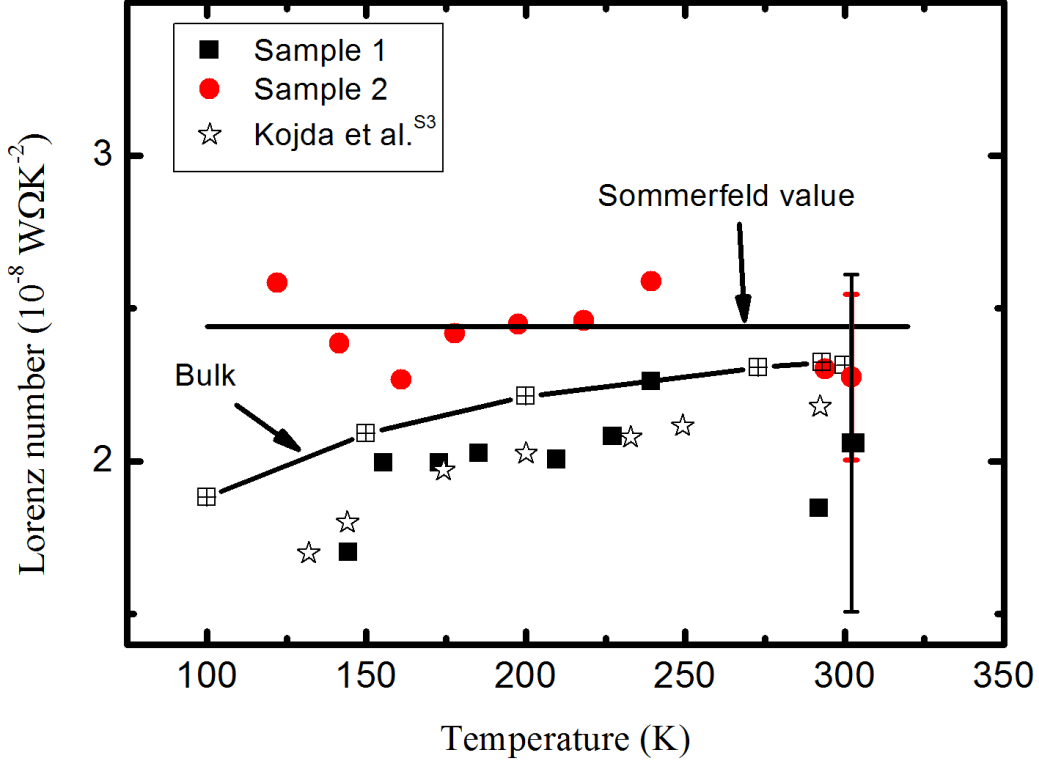


Figure S6. Lorenz number of the two samples as a function of temperature when *R*_s_=0.2 m K W^-1^.

**References**

^S1^ Gall, D. Electron mean free path in elemental metals. J. Appl. Phys. **119,** 085101(2016).

^S2^ Yu, C. *et al.* Thermal contact resistance and thermal conductivity of a carbon nanofiber, J. Heat Trans. **128**, 234-239 (2006).

^S3^ Kojda, D. *et al.* Temperature-dependent thermoelectric properties of individual silver nanowires. Phys. Rev. B **91**, 024302 (2015).
